# Supplementary figures and images for: In Vitro Antiviral Activity of Rhodiola crenulata Extract against Zika Virus and Japanese Encephalitis Virus: Viral Binding and Stability
Source: Pharmaceuticals (Basel). 2024 Jul 26;17(8):988. doi: 10.3390/ph17080988 (PMC11357342; doi:10.3390/ph17080988)

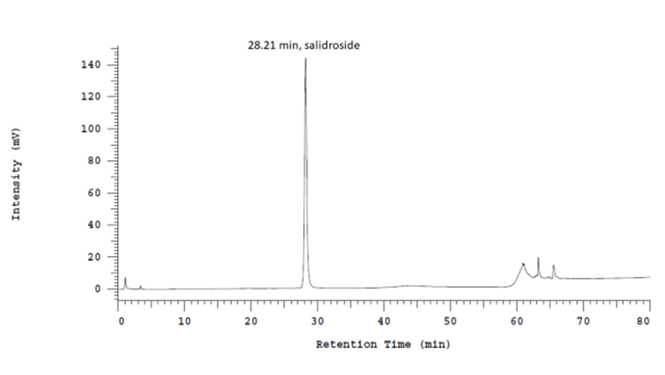

Supplement: Supplementary file 1 [file pharmaceuticals-17-00988-s001.zip › Figure S1A.tif]

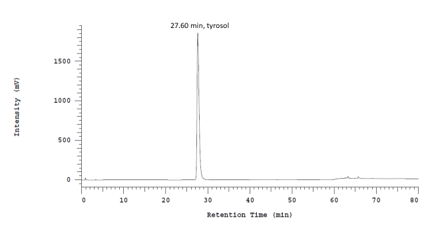

Supplement: Supplementary file 1 [file pharmaceuticals-17-00988-s001.zip › Figure S1B.tif]

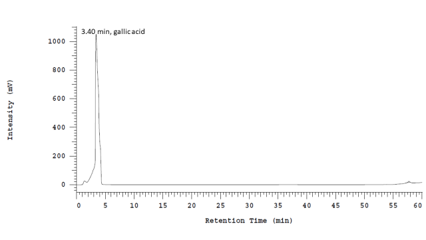

Supplement: Supplementary file 1 [file pharmaceuticals-17-00988-s001.zip › Figure S1C.tif]

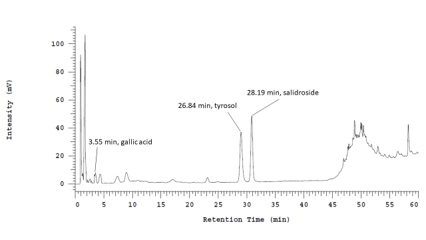

Supplement: Supplementary file 1 [file pharmaceuticals-17-00988-s001.zip › Figure S1D.tif]

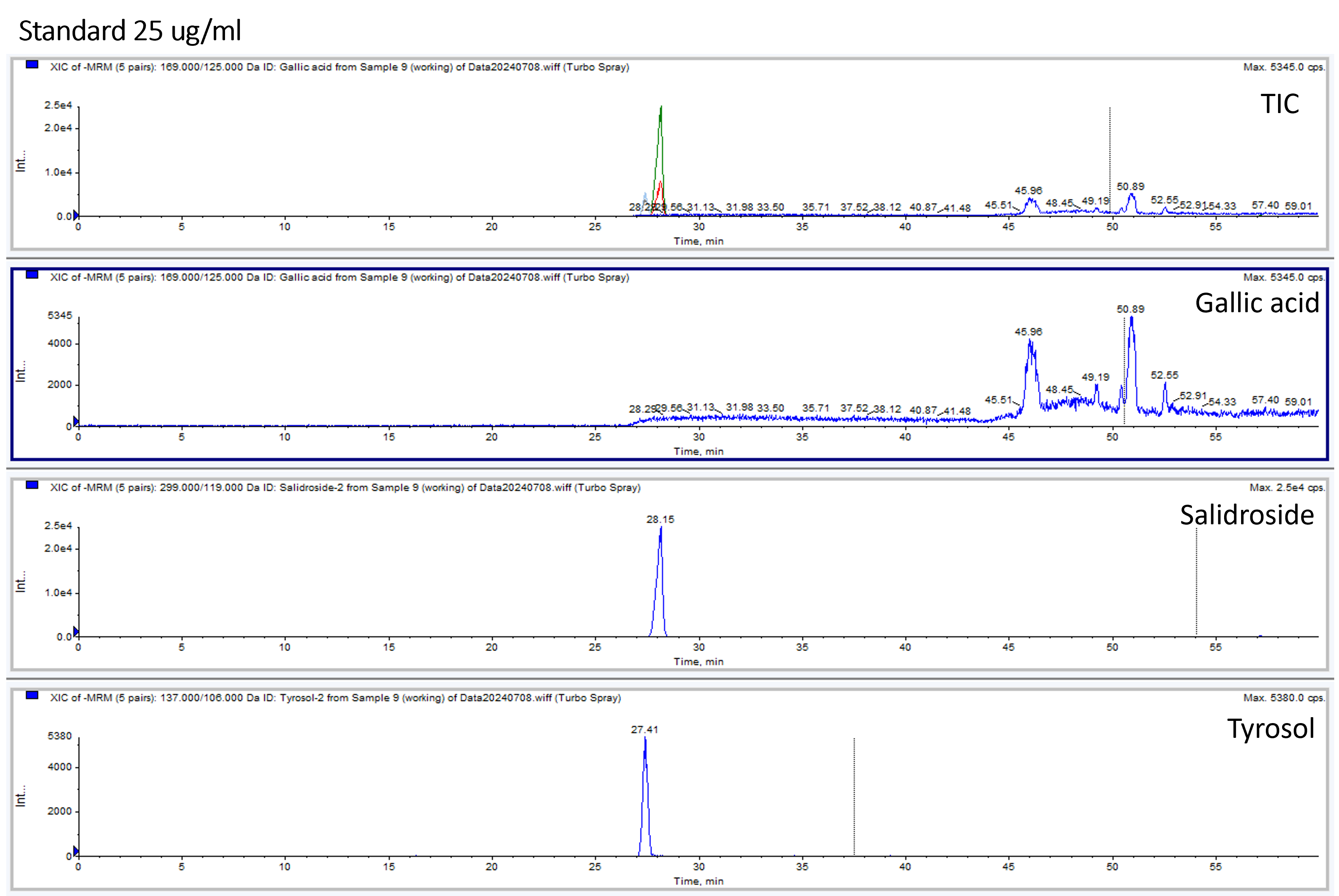

Supplement: Supplementary file 1 [file pharmaceuticals-17-00988-s001.zip › Figure S2A.tif]

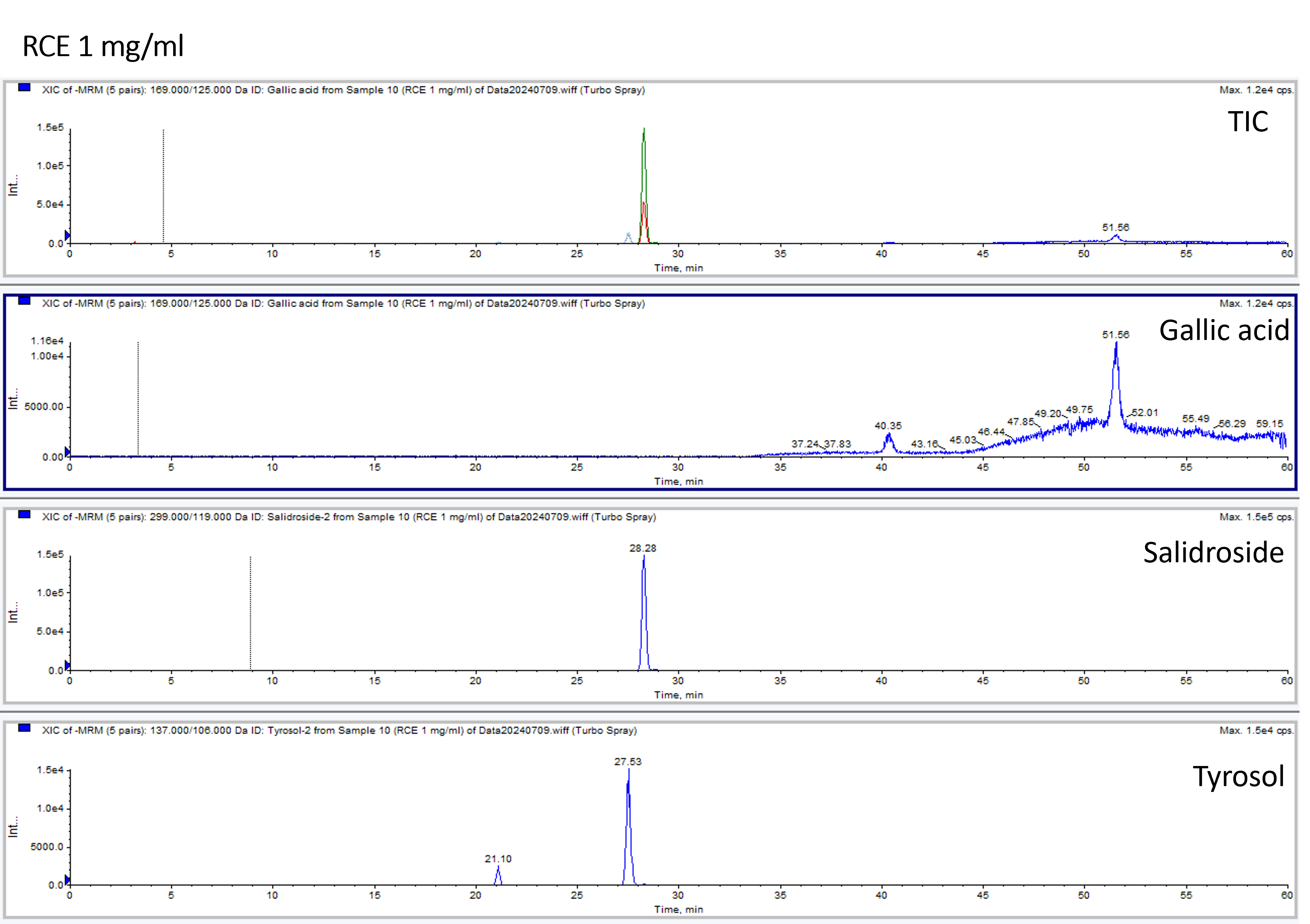

Supplement: Supplementary file 1 [file pharmaceuticals-17-00988-s001.zip › Figure S2B.tif]

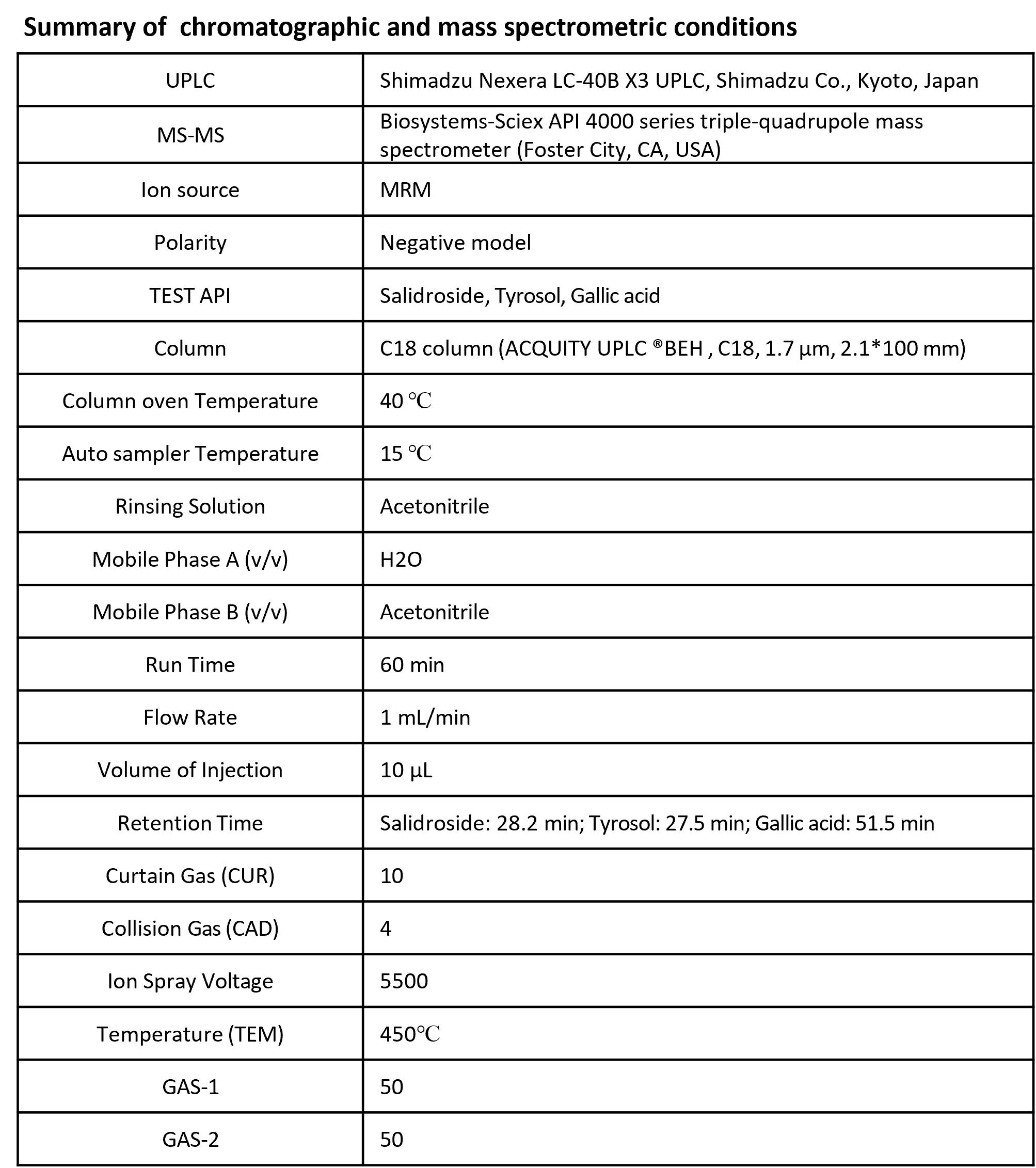

Supplement: Supplementary file 1 [file pharmaceuticals-17-00988-s001.zip › Figure S2C.tif]

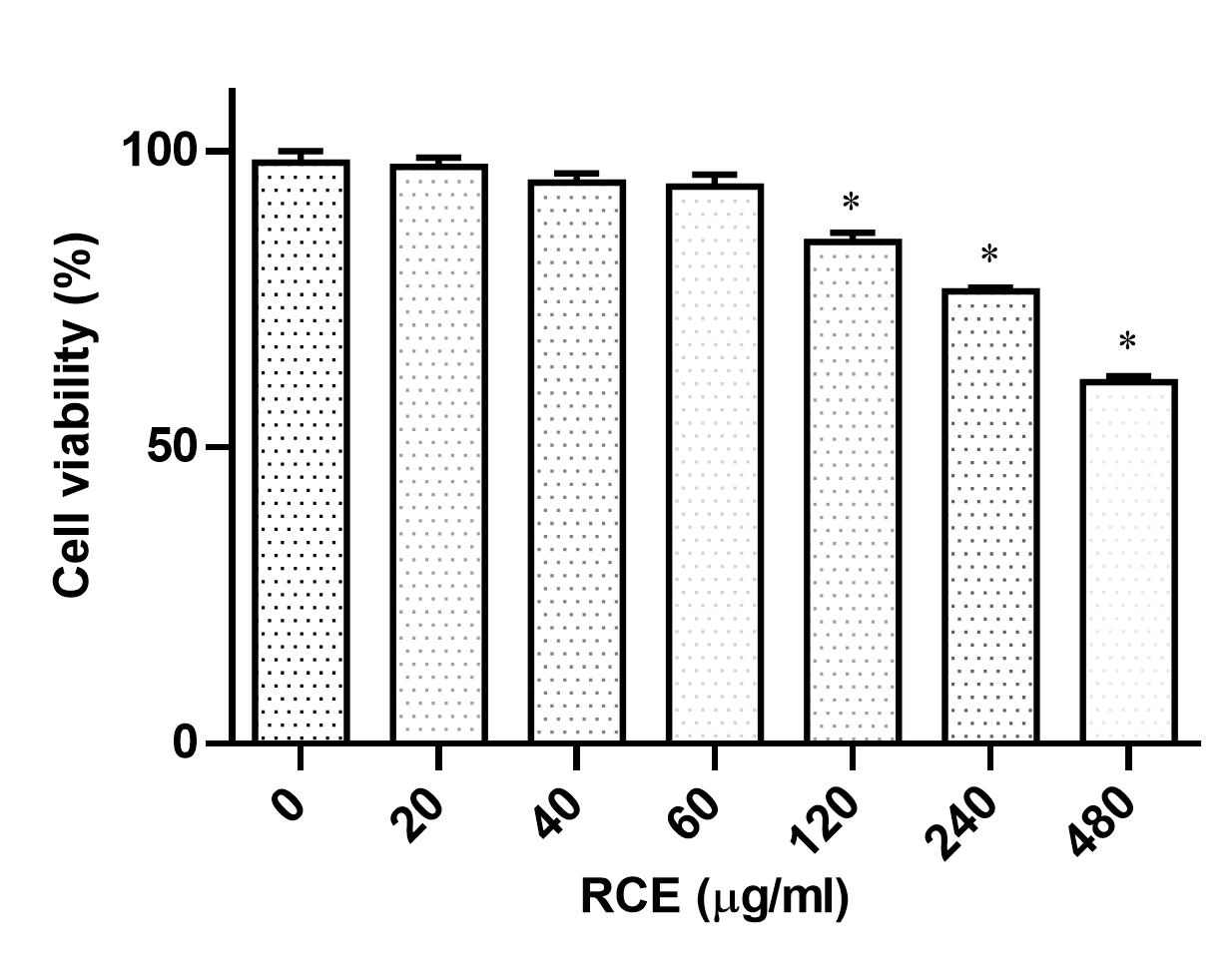

Supplement: Supplementary file 1 [file pharmaceuticals-17-00988-s001.zip › Figure S3A.tif]

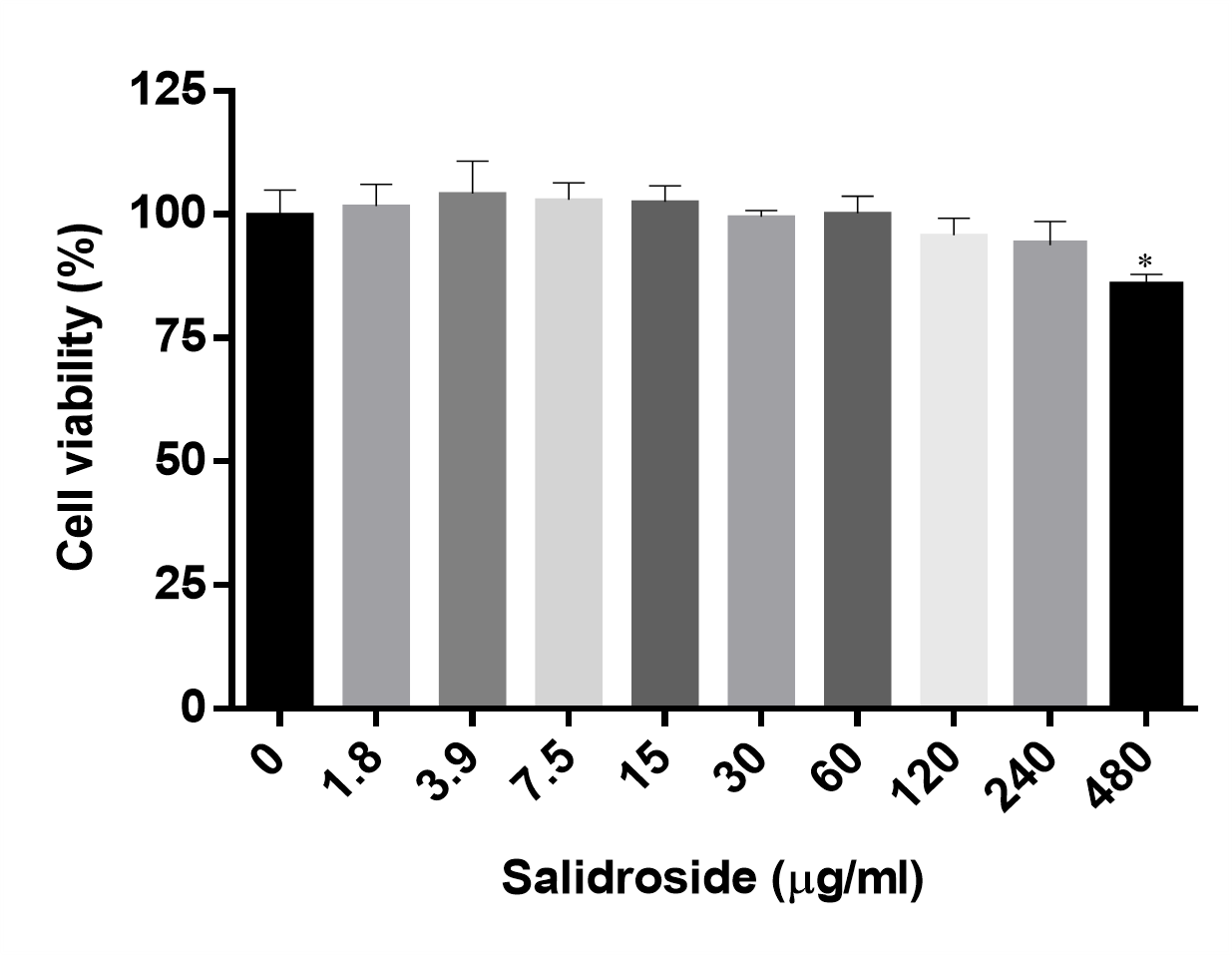

Supplement: Supplementary file 1 [file pharmaceuticals-17-00988-s001.zip › Figure S3B.tif]

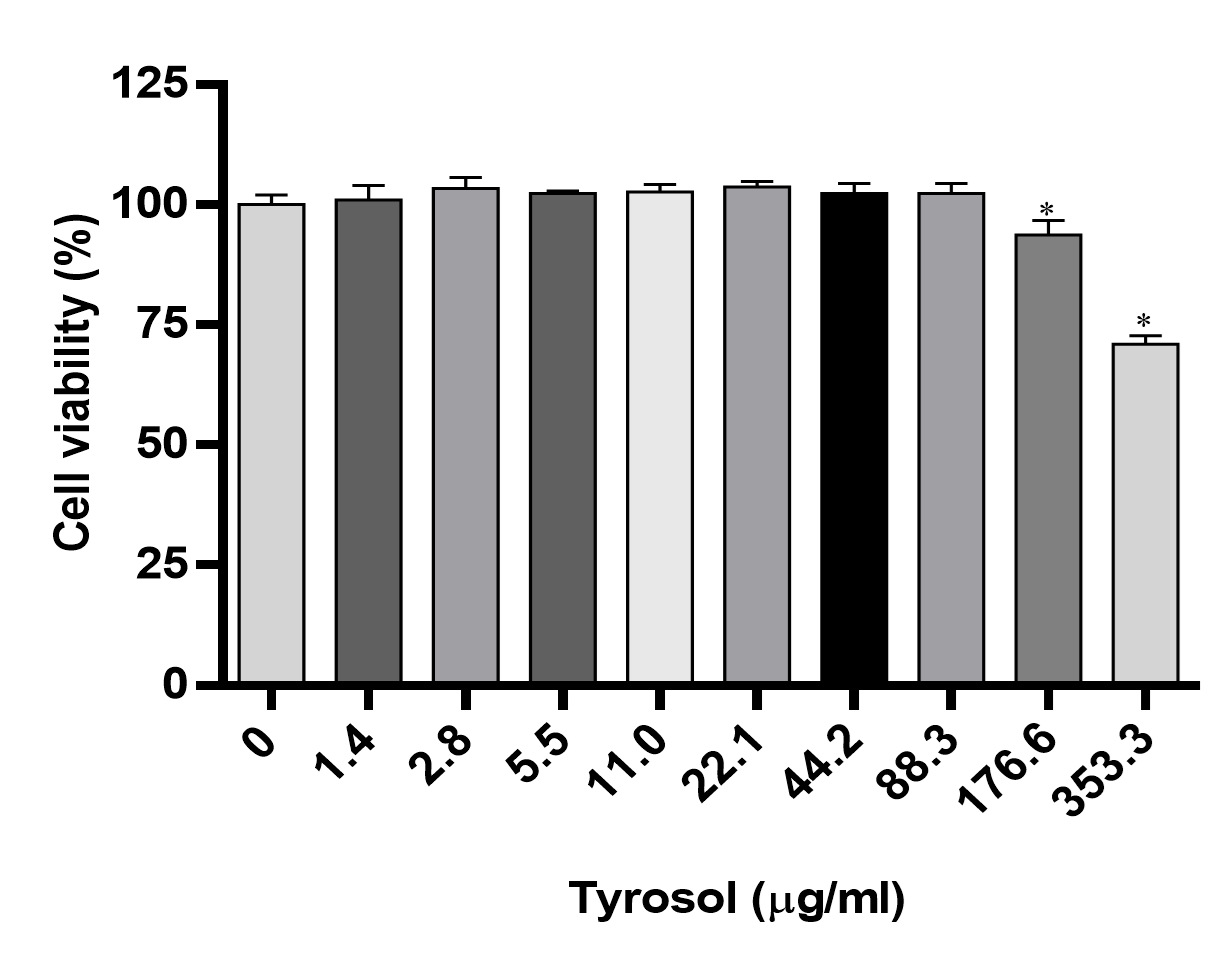

Supplement: Supplementary file 1 [file pharmaceuticals-17-00988-s001.zip › Figure S3C.tif]

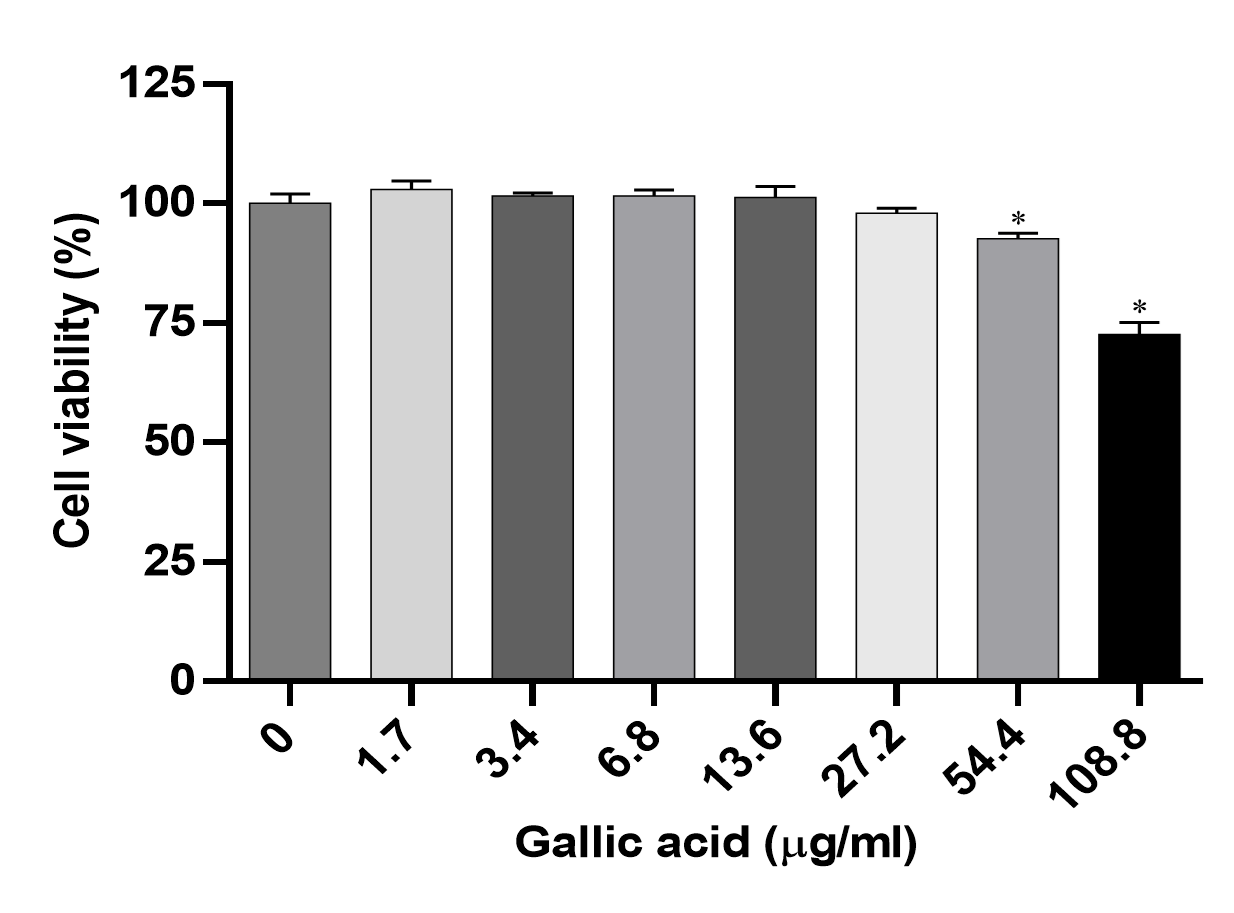

Supplement: Supplementary file 1 [file pharmaceuticals-17-00988-s001.zip › Figure S3D.tif]
